# Highly frequent *PIK3CA *amplification is associated with poor prognosis in gastric cancer

**DOI:** 10.1186/1471-2407-12-50

**Published:** 2012-02-01

**Authors:** Jing Shi, Demao Yao, Wei Liu, Na Wang, Hongjun Lv, Guanjun Zhang, Meiju Ji, Li Xu, Nongyue He, Bingyin Shi, Peng Hou

**Affiliations:** 1 Department of Endocrinology, The First Affiliated Hospital of Xi'an Jiaotong University School of Medicine, Xi'an 710061, the People's Republic of China; 2 Department of Surgery, The First Affiliated Hospital of Xi'an Jiaotong University School of Medicine, Xi'an 710061, the People's Republic of China; 3 Department of Pathology, The First Affiliated Hospital of Xi'an Jiaotong University School of Medicine, Xi'an 710061, the People's Republic of China; 4 State Key Laboratory of Bioelectronics, Southeast University, Nanjing 210096, the People's Republic of China

**Keywords:** Gastric cancer, PI3K/Akt pathway, *PIK3CA *mutations, *PIK3CA *amplification, Poor survival

## Abstract

**Background:**

The phosphoinositide 3-kinase (PI3K)/Akt pathway plays a fundamental role in cell proliferation and survival in human tumorigenesis, including gastric cancer. *PIK3CA *mutations and amplification are two major causes of overactivation of this pathway in human cancers. However, until this work, there was no sound investigation on the association of *PIK3CA *mutations and amplification with clinical outcome in gastric cancer, particularly the latter.

**Methods:**

Using direct sequencing and real-time quantitative PCR, we examined *PIK3CA *mutations and amplification, and their association with clinicopathological characteristics and clinical outcome of gastric cancer patients.

**Results:**

*PIK3CA *mutations and amplification were found in 8/113 (7.1%) and 88/131 (67%) gastric cancer patients, respectively. *PIK3CA *amplification was closely associated with increased phosphorylated Akt (p-Akt) level. No relationship was found between *PIK3CA *mutations and clinicopathological characteristics and clinical outcome in gastric cancer. *PIK3CA *amplification was significantly positively associated with cancer-related death. Importantly, Kaplan-Meier survival curves revealed that the patients with *PIK3CA *amplification had significantly shorter survival times than the patients without *PIK3CA *amplification.

**Conclusions:**

Our data showed that *PIK3CA *mutations were not common, but its amplification was very common in gastric cancer and may be a major mechanism in activating the PI3K/Akt pathway in gastric cancer. Importantly, Kaplan-Meier survival curves revealed that *PIK3CA *amplification was significantly positively associated with poor survival of gastric cancer patients. Collectively, the PI3K/Akt signaling pathway may be an effective therapeutic target in gastric cancer.

## Background

Gastric cancer is highly prevalent in Asia, particularly China, and is one of the leading cause of cancer-related death worldwide [1]. The histological classifications of gastric cancer involve two distinct types, intestinal and diffuse [2]. Although recent diagnostic and therapeutic advances have provided excellent survival for patients with early gastric cancer, the gastric cancer is usually diagnosed at an advanced stage and the prognosis is still poor [3], reflecting limited advances in our understanding of the pathogenesis of this disease and the molecular events that contributed to its development. A better understanding of the molecular mechanisms of gastric cancer may lead to new diagnostic, therapeutic and preventive approaches to this disease.

Gastric cancer is chronic proliferative disease characterized by multiple genetic and epigenetic events [4-6]. The aberrant signaling of major pathways is involved in this process, including phosphoinositide 3-kinase (PI3K)/Akt pathway, which plays a fundamental role in cell proliferation and survival in gastric tumorigenesis [7-9]. A key step in the signaling of PI3K/Akt pathway is the generation of phosphatidylinositol-3,4,5-trisphosphate (PIP3) catalyzed by PI3K. PI3K is composed of heterodimers of a p85 regulatory subunit and one of the several p110 catalytic subunits. Among several isoforms of the catalytic subunits, only the α-type, PIK3CA, has been shown to harbor oncogenic mutations in human cancer [10-12], including gastric cancer [13-15], implying an important role of *PIK3CA *mutations in gastric carcinogenesis. In addition to mutations, genomic amplification of *PIK3CA *has been reported in various human cancers, including ovarian cancer, cervical cancer, thyroid cancer, and non-small cell lung cancer (NSCLC) [16-19]. Increased copy number of *PIK3CA *was closely associated with elevated mRNA or protein expression [16-18]. Importantly, *PIK3CA *overexpression caused by gene amplification increased PI3-kinase activity and phosphorylated Akt level, which was associated with aberrant cell proliferation and apoptosis, both of which are directly linked to tumorigenesis [16,17,20].

In the present study, we analyzed a large cohort of clinically well-characterized gastric cancers for the presence of mutations in the exons 9 and 20 of *PIK3CA *gene by direct sequencing and *PIK3CA *amplification by real-time quantitative PCR, and correlated the presence of *PIK3CA *mutations and amplification with clinicopathological characteristics and clinical outcome of gastric cancer patients.

## Methods

### Clinical samples

With the institutional review board approval, a total of 131 paraffin-embedded gastric cancer tissues were randomly obtained from the First Affiliated Hospital of Xi'an Jiaotong University School of Medicine. The 37 normal controls from the patients with chronic gastritis who underwent endoscopic biopsy, were also obtained from the First Affiliated Hospital of Xi'an Jiaotong University School of Medicine. None of these patients received chemotherapy and radiotherapy before the surgery. Informed consent was obtained from each patient before the surgery. All of the samples were histologically examined by a pathologist at Department of Pathology of the Hospital to identify the clinicopathologic characteristics of the tumors, which are shown in Table 1.

**Table 1:** Association of *PIK3CA *mutations and amplification with clinicopathologic variables

Variable	*PIK3CA *mutations (n = 113)			*PIK3CA *amplification (n = 131)		
	
	Yes	No	*P*	Yes	No	*P*
No.of patients	8	105		88	43	

Gender						

Male	5	82	0.57	68	34	0.82

Female	3	23		20	9	

Age, years						

Mean	55.5	59.1	0.47	58.6	61.4	0.25

SD	19.8	12.7		13.3	12.1	

Tumor localization						

gastric cardia	0	26	0.98	23	12	0.66

gastric body	5	24		22	12	

gastric antrum	3	55		43	19	

Tumor size (cm^3^)						

≤ 3	3	35	0.68	29	14	0.88

3-5	3	35		32	15	

> 5	2	35		27	14	

Differentiation						

well/moderate	1	44	0.21	33	23	0.08

poor/undifferentiation	7	61		55	20	

Tumor invasion						

T1	2	14	0.96	9	5	0.59

T2	0	17		15	8	

T3	6	72		62	30	

T4	0	2		2	0	

TNM stage						

I	2	27	0.91	20	10	0.86

II	1	14		15	6	

III	5	58		49	25	

IV	0	6		4	2	

Residual tumor						

Yes	1	12	1.00	12	2	0.10

No	7	93		76	41	

Lymph node metastasis (LNM)						

Yes	5	65	1.00	57	24	0.32

No	3	40		31	19	

No. of LNM						

N0	3	40	0.63	31	19	0.20

N1 (1-6)	4	36		32	16	

N2 (7-15)	1	23		20	7	

N3 (≥ 16)	0	6		5	1	

Survival status						

Dead	3	53	0.73	51	15	0.01*

Alive	5	52		37	28	

* Significant at *P *< 0.05

### Tissues and DNA preparation

Serial sections from each tumor sample were cut. One section (5 μm) was stained by hematoxylin and eosin (H&E) and a tumor representative tissue was marked by an expert surgical pathologist for gastric cancer. The next section (8 μm) was deparaffinized and stained with hematoxylin. Tumor tissues were isolated by manual microdissection under an inverted microscope using the marked H&E section for target tissue identification. Genomic DNA was extracted from isolated tissues as previously described [18]. Briefly, after a treatment for overnight at room temperature with xylene to remove pareffin, tissues were digested with 1% sodium dodecyl sulfate (SDS) and 0.5 mg/ml proteinase K at 48°C for 48 h, with addition of several spiking aliquots of concentrated proteinase K to faciliate digestion. DNA was subsequently isolated using standard phenol/chloroform protocol, and was dissolved in distilled water and stored at -80°C until use. Subsequent tissue sections (5 μm) were prepared on 3-aminopropyltriethoxysilane (APTES) coated slides for immunohistochemical assay.

### Mutation analysis of *PIK3CA *gene

In the present study, we selected exon 9 (for the regulatory helical domain) and exon 20 (for the kinase domain) of *PIK3CA *gene, two mutational hotspot regions, for mutation analysis as previous large-sclae analysis of *PIK3CA *mutations in various human cancers revealed that > 80% of the mutations clustered within these domains [10,13,21,22]. Genomic DNA was amplified by PCR using the amplifying and sequencing primers for these exons of *PIK3CA *gene as described previously [10]. The PCR was performed in a final volume of 20 μl on a 96-well plate, which containing~60 ng genomic DNA, 16.6 mM ammonium sulfate, 67 mM Tris (pH 8.8), 5% dimethylsulfoxide, 2 mM MgCl_2_, 10 mM 2-mercaptoethanol, 200 μM of each deoxynucleotide triphosphate (dATP, dCTP, dGTP, and dTTP), 200 nM of each primer (forward and reverse), and 0.6 U platinum DNA *Taq *polymerase (Invitrogen Life Technologies, Inc., Gaithersburg, MD). Step-down PCR was run in a Thermal cycler (Bio-Rad Laboratories, Inc., CA) as follows: after a 4-min denaturing at 95°C, the PCR was run with each temperature for 1 min at six step-down steps, for two cycles each. The denaturing temperature was 95°C, and extension temperature was 72°C for each step, with the annealing temperature of 66°C, 64°C, 62°C, 60°C, 58°C, and 56°C from the first to the last step. The PCR was finally run at 95°C, 56°C, and 72°C each for 1 min for 35 cycles, followed by an elongation at 72°C for 5 min. The PCR products were electrophoresed on a 1.2% agarose gel and visualized under UV illumination using an ethidium bromide stain. The direct sequencing was performed to analyze *PIK3CA *mutations on an ABI PRISM 3700 DNA Analyzer (Applied Biosystems) at the sequencing core of Beijing Genomics Institute (BGI, Beijing).

### Copy number analysis of *PIK3CA *gene with real-time quantitative PCR

We analyzed the copy number of *PIK3CA *gene in 131 gastric cancer samples and 37 controls by real-time quantitative PCR technique on a CFX384 Thermal Cycler Dice™ real-time PCR system (Bio-Rad Laboratories, Inc., CA) as described previously [23]. This method was well established and validated by florescence *in situ *hybridization (FISH) [23,24], which has been widely used in the various human cancers [18,23-25]. Specific primers and TaqMan probes were designed using Primer Express 3.0 (Applied Biosystems) to amplify *PIK3CA *and *β-actin *genes as described previously [18]. Using a PCR protocol described previously [23], each sample was run in triplicate, and *β-actin *was run in parallel to standardize the input DNA. Standard curves were established using serial dilutions of normal leukocyte DNA with a quantity range of 3.75-60 ng per 2 μl. Copy gain (or amplification) of *PIK3CA *gene was defined by a copy number ≥ 4.

### Immunohistostaining (IHS) of phosphorylated akt (p-akt)

This procedure was pursued to investigate the level of p-Akt in relation to *PIK3CA *copy gain in the tumor. Briefly, formaldehyde fixed, paraffin-embedded tissue sections (5 μm) were deparaffinized and rehydrated in xylene and degradation alcohol. Antigen unmasking was performed by pretreatment of the slides in 0.01 M citrate buffer (pH 6.0) at 98°C for 15 min using a microwave oven. The slides were then cooled to room temperature on bench top for 20 min. Endogenous peroxidase was cleaned by incubating the slides in 3% hydrogen peroxide for 5 min. After washed in 0.01 M PBS (pH 7.4), the sections were incubated for 10 min at room temperature with normal goat serum, followed by incubation with anti-p-Akt antibody (BS4007, Bioworld Technology, Inc., MN) overnight at 4°C. The sections were subsequently washed with PBS and incubated with biotinylated goat anti-rabbit IgG (SP9000, Zhongshan Goldenbridge, Beijing) and streptavidin-peroxidase complex, followed by reaction with diaminobenzidine and counterstaining with hematoxylin. Negative control was performed by omission of primary antibody. For positive control, we used samples from previously examined gastric cancer tissues positive for p-Akt. The level of p-Akt was scored in double-blinding way (i.e., without knowing the *PIK3CA *copy number of the case), and 0, 1, 2, 3 reprints negative, weak positive, positive, and strong positive, respectively.

### Statistical analysis

The Mann-Whitney *U *test was used to compare copy number of *PIK3CA *gene between gastric cancer and normal gastric samples. Correlation between *PIK3CA *mutations or amplification and clinicopathological characteristics was analyzed by Fisher's exact test or Pearson's Chi square test. The Mann-Whitney *U *test was used for ordinal variables. Factors (*PIK3CA *mutations or amplification) associated with clinicopathological characteristics of tumor were assessed univariately using the SPSS statistical package (11.5, Chicago, IL, USA). Multivariate models were then developed that adjusted for the most important covariates, including tumor size, differentiation, tumor stage, lymph node metastasis and survival status. Survival length was determined from the day of primary tumor surgery to the day of death or last clinical follow-up. The Kaplan-Meier method was used for survival analysis grouping with *PIK3CA *mutations or amplification. Differences between curves were analyzed using the log-rank test. Multivariate Cox regression analysis was used to evaluate the effect of *PIK3CA *amplification on survival of independently of age, differentiation, lymph node metastasis, and TNM stage. All statistical analyses were performed using the SPSS statistical package (11.5, Chicago, IL, USA). *P *values < 0.05 were considered significant.

## Results

### *PIK3CA *mutations and amplification in gastric cancer

**Figure 1:**
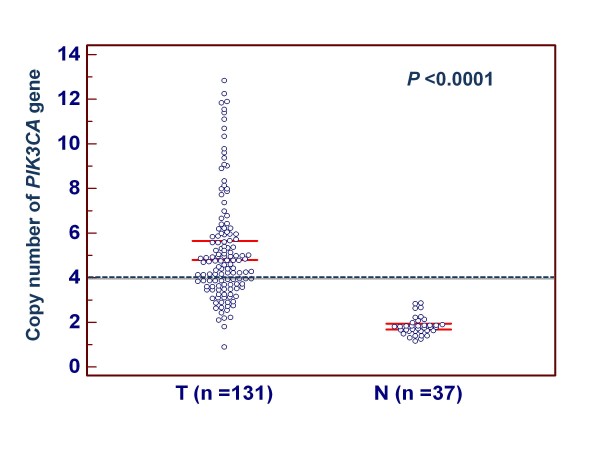
**Copy number of the *PIK3CA *gene corresponding to each individual case of gastric cancers and normal gastric tissues (*circle*)**. Real-time quantitative PCR was performed to analyze the copy number of *PIK3CA *gene in a large cohort of gastric cancers and normal gastric tissues. Details are as described in Methods. Horizonal lines indicate a 95% confidence interval for the sample mean. T, tumor tissues; N, normal gastric tissues.

As the first step to understand the role of *PIK3CA *gene in gastric cancer, we sequenced exons 9 and 20 of this gene in a large cohort of gastric cancers. A total of 8 *PIK3CA *mutations (7.1%) were found in the 113 gastric cancers. Of these, 3 mutations, including P539S, E542K and E545K, were in the exon 9, and 5 mutations, including H1048D,G1050S,W1057R,W1057C and I1062T, were in the exon 20. All mutations found were heterozygous missense single base substitutions (see Additional file 1: Figure S1). To analyze copy number of *PIK3CA *gene, real-time quantitative PCR assay was performed in the 131 gastric cancers and 37 normal controls. With a gene copy number of 4 or more defined as amplification, we found the incidence of *PIK3CA *amplification in gastric cancers was 67% (88/131) in the present study, whereas no *PIK3CA *amplification was found in the 37 normal controls. Copy number of *PIK3CA *gene corresponding to each individual case of gastric cancers and normal gastric tissues is shown in Figure 1. Statistical analysis showed that copy number of *PIK3CA *gene in gastric cancers was significantly higher than normal gastric tissues (*P *< 0.0001).

**Figure 2:**
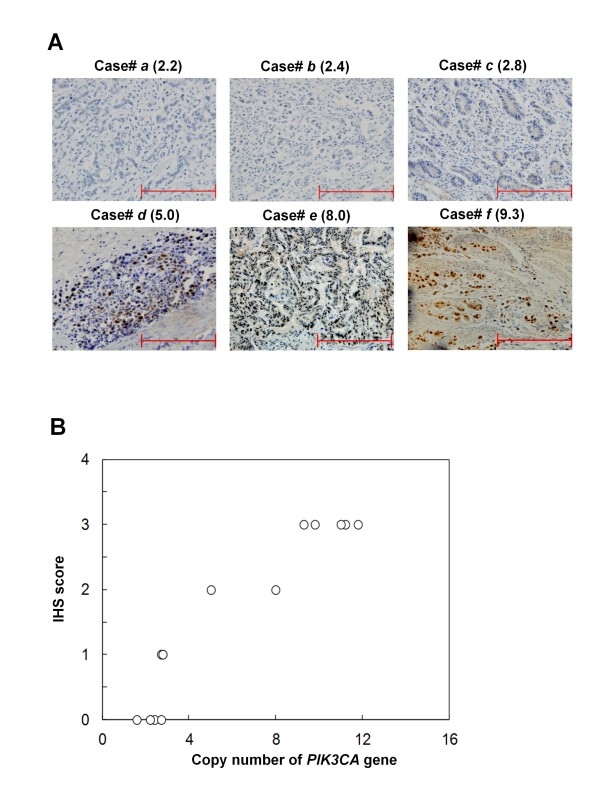
**Immunohistostaining of phosphorylated Akt (p-Akt): correlation of *PIK3CA *copies with increased p-Akt level**. (**A**) Representative samples of immunohistostaining on gastric cancer histologic slides using anti-p-Akt antibodies. Increasing extent of specific staining (*brown color*) in association with increasing *PIK3CA *copies (*number inside bracket*s). (**B**) Association of immunohistostaining (IHS) score of p-Akt with *PIK3CA *copies on 13 randomly selected gastric cancer samples.

To investigate the effect of *PIK3CA *amplification on the activity of PI3K/Akt signaling pathway, we randomly selected 13 gastric cancer samples with various *PIK3CA *copies and did immunohistostaining for p-Akt. As illustrated by the representative samples in Figure 2A, and all of selected 13 samples in Figure 2B, increased staining of p-Akt was seen with increased *PIK3CA *copies.

### Association of *PIK3CA *mutations and amplification with clinicopathological characteristics of gastric cancer

Because *PIK3CA *mutations and amplification, particularly highly frequent *PIK3CA *amplification, was demonstrated in gastric cancer, the association of *PIK3CA *mutations and amplification with clinicopathological characteristics was analyzed in a large cohort of clinically well-characterized gastric cancers. As shown in Table 1, *PIK3CA *mutations and amplification, particularly the former, showed no relation to most of clinicopathological characteristics. *PIK3CA *amplification was significantly positively associated with cancer-related death (*P *= 0.01). Although no statistical significance was noted, there was a positive association of *PIK3CA *mutations and amplification with tumor differentiation (Table 1). Similarly, the univariate analysis also showed *PIK3CA *amplification was associated with a significantly increased risk of cancer-related death (OR = 2.57, 95% CI = 1.21-5.48; *P *< 0.05), and there was a positive association of *PIK3CA *mutations (OR = 5.05, 95% CI = 0.60-42.5) and *PIK3CA *amplification (OR = 1.92, 95% CI = 0.92-4.01) with tumor differentiation (see Additional file 1: Table S1). In order to assess the independent association of *PIK3CA *mutations and amplification with tumor size, tumor differentiation, tumor stage, lymph node metastasis and survival status, we conducted two multivariable logistic regressions (Table 2). Similar to univariate analysis, after adjustment, *PIK3CA *amplification was still significantly associated with higher mortality (OR = 3.50, 95% CI = 1.36-9.00; *P *< 0.05), and *PIK3CA *mutations remained positively associated with tumor differentiation (OR = 5.53, 95% CI = 0.65-47.4) (Table 2).

**Table 2:** *PIK3CA *mutations and amplification in gastric cancer--multivariable models assessing tumor size, differentiation, tumor stage, lymph node metastasis and survival status (OR^† ^and 95%CI)

Factors	*PIK3CA *mutations [OR^† ^(95% CI)]	*PIK3CA *amplification [OR^† ^(95% CI)]
Tumor size^1^	0.80 (0.29-2.21)	0.88 (0.50-1.54)

Differentiation^2^	5.53 (0.65-47.4)	1.85 (0.86-4.02)

Tumor stage^3^	1.19 (0.33-4.35)	0.60 (0.31-1.17)

Lymph node metastasis	1.32 (0.13-13.6)	1.44 (0.45-4.58)

Survival status^4^	0.41 (0.07-2.41)	3.50 (1.36-9.00)*

* Significant at *P *< 0.05

† OR: odds ratio with 95% confidence interval

^1 ^Tumor size (≤ 3 cm; > 3 cm and ≤ 5 cm; > 5)

^2 ^Differentiation (well or moderate; poor or no differentiation)

^3 ^Tumor stage (I; II; III; IV)

^4 ^Survival status (alive; dead)

### Effect of *PIK3CA *mutations and amplification on poor survival in gastric cancer

The Kaplan-Meier estimator of the survivorship function was used to determine the effect of *PIK3CA *mutations and amplification on the survival of gastric cancer patients. The overall survival of gastric cancer patients with and without *PIK3CA *mutations/amplification was compared using the log-rank test. As shown in Additional file 1: Figure S2, *PIK3CA *mutations did not affect the overall prognosis of gastric cancer patients (*P *= 0.44). However, the patients with *PIK3CA *amplification had significantly shorter survival times than the patients without *PIK3CA *amplification (516.0 months vs. 758.4 months on average; *P *= 0.008).

**Figure 3:**
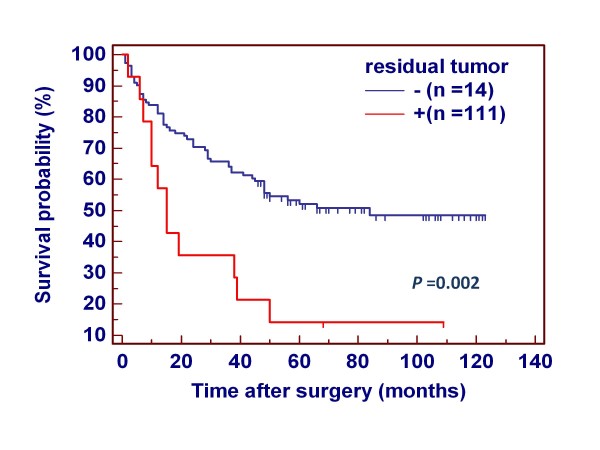
**Effect of residual tumor after surgery on poor survival in gastric cancer**. Clinical outcome was evaluated according to the presence of residual tumor after surgery in a number of gastric cancers. Kaplan-Meier survival curves show that the patients with residual tumor after surgery had significantly shorter survival times than the patients without residual tumor (*P *= 0.01). +, the patients with residual tumor after surgery; -, the patients without residual tumor after surgery.

**Figure 4:**
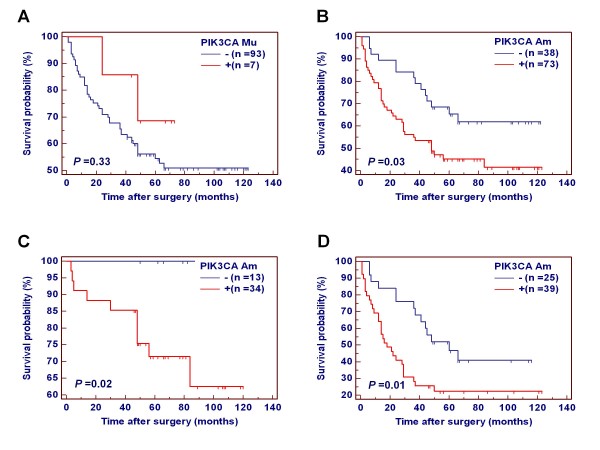
**Association of *PIK3CA *mutations and amplification with clinical outcome in patients with gastric cancer**. Kaplan-Meier analysis of survival was performed according to the presence of *PIK3CA *mutations or amplification in a large cohort of gastric cancers. (**A**) Kaplan-Meier survival curves show that *PIK3CA *mutations were not associated with poor survival of the patients. (**B**) The patients with *PIK3CA *amplification had poorer survival than the patients without *PIK3CA *amplification. (**C**) *PIK3CA *amplification was extremely significantly associated with poor survival in the patients who had early-stage tumors (*P *= 0.004). (**D**) *PIK3CA *amplification was marginally significantly associated with poor survival in the patients who had late-stage tumors (*P *= 0.06). *PIK3CA *Mu, *PIK3CA *mutations; *PIK3CA *Am, *PIK3CA *amplification; +, harboring *PIK3CA *mutations or amplification; -, the lack of *PIK3CA *mutations or amplification.

Many evidences showed that residual tumor after surgery is an independent risk factor for gastric cancer patients. In the present study, indeed, the patients with residual tumor after surgery had significantly shorter survival times than the patients without residual tumor (343.2 months vs. 627.6 months on average; *P *= 0.002) (Figure 3). Thus, we excluded the patients with residual tumor to explore the association of *PIK3CA *mutations and amplification with the survival of gastric cancer patients again. Similar to the findings in Additional file 1: Figure S2, *PIK3CA *mutations did not have any prognostic value for gastric cancer patients (Figure 4A). However, *PIK3CA *amplification significantly affected clinical outcomes of gastric cancer patients. The patients with *PIK3CA *amplification had significantly shorter survival times than the patients without *PIK3CA *amplification (540.0 months vs. 794.4 months on average; *P *= 0.03) (Figure 4B). The data were stratified further according to the TNM tumor stage, because it is an independent risk factor in gastric cancer patients. As shown in Figure 4C and 4D, *PIK3CA *amplification was significantly associated with poor survival whatever the patients who had early-stage tumors (stage I and II) or late-stage tumors (stage III and IV). Multivariate Cox regression analysis indicated that *PIK3CA *amplification is a predictor of poor prognosis for gastric cancer patients (HR = 2.59, 95% CI = 1.39-4.82, *P *= 0.003) as an independently variable with respect to age, differentiation, lymph node metastasis, and TNM stage (Table 3).

**Table 3:** Multivariate Cox regression analysis of clinical variables on overall survival

Variable	HR^†^	95% CI	*P*
*PIK3CA *amplification	2.59	1.39-4.82	0.003

Age^1^	1.28	0.98-1.67	0.07

Differentiation^2^	1.16	0.66-2.05	0.61

Lymph node metastasis^3^	2.70	1.01-7.21	0.047

TNM stage^4^	2.07	1.17-3.65	0.01

† HR: Hazard Ratio

^1 ^Age (per 10 years)

^2 ^Differentiation (well or moderate; poor or no differentiation)

^3 ^Lymph node metastasis (Yes vs. No)

^4 ^Tumor stage (I; II; III; IV)

## Discussion

It has been well documented that the PI3K/Akt pathway plays an important role in cancer-related functions of cell proliferation, catabolism, cell adhesion and apoptosis [10,16,17,26,27], and it has a crucial role in the tumorigenesis and pathogenesis of many human cancers. The previous studies have shown that *PIK3CA*, as a subunit of PI3K, is frequently mutated in various human cancers, such as ovarian, thyroid cancer, breast, cervical cancers, and pituitary tumors [10,12,13,16,17,21,28]. However, our study showed that the most common activating *PIK3CA *mutations reported in other cancers were not frequent in gastric cancer. Therefore, the *PIK3CA *mutations may not be a common mechnaism in the activation of PI3K/Akt signaling pathway. Instead, our study demonstrated *PIK3CA *gene was highly amplified in gastric cancer. Genomic amplification, rather than gene mutations, may represent another major signature of neoplastic transformation and tumor progression [29]. Chromosome copy number abnormalities have been frequently identified in gastric cancer [30], including *PIK3CA *amplification [31].

Of particular interest was the *PIK3CA *amlification was closely associated with elevated p-Akt, suggesting that this genetic alteration could lead to oncogenic activation of PI3K/Akt signaling pathway and thus contributed to the malignant progression of gastric cancer. It was consistent with a previous study [31], which *PIK3AC *gene was aberrantly amplified, and mutually excluded with monallelic deletion of *PTEN *gene in gastric cancer, further supporting that *PIK3CA *amplification, like *PTEN *loss, might contribute to gastric tumorigenesis through the activation of the PI3K/Akt pathway.

Given *PIK3CA *mutations and amplification play the critical role in gastric tumorigenesis, we investigated their clinical significances and prognostic values in a large cohort of gastric cancer patients who had known survival data. Out data showed that *PIK3CA *mutations were not associated with most of clinicopathological characteristics and clinical outcome in gastric cancer. One possibility is the limited number of *PIK3CA *mutations found in this study. However, *PIK3CA *amplification was associated with a significantly increased risk of cancer-related death, and positively associated tumor differentiation. Most noteworthy, *PIK3CA *amplification significantly affected the overall prognosis in gastric cancer whatever the patients who had early-stage or late-stage tumors, suggesting that this genetic event plays an important role in the multistep process of gastric carcinogenesis. Taken together, *PIK3CA *amplification may be served as a potential prognostic marker for gastric cancer patients.

The prognostic markers may have another role in predicting and guiding the clinical treatment of cancer patients by allowing the identification of patients suited to current therapies. In this era of molecularly targeted therapy, inhibitors and antibodies targeting specific molecules are vigorously being developed, and some have been demonstrated to be effective in clinical settings, such as hematological malignancies [32] and non-small cell lung cancer (NSCLC) [33,34]. Of interest, some of these targeted drugs are more effective against the genetically altered cancerous form of the target, as illustrated by the activities of gefitinib and erlotinib against the mutated EGFR present in NSCLC [33,34], and the activity of trastuzumab against breast cancer with amplified ErbB2 [35]. Mutations and amplification of certain kinases are involved in gastric tumorigenesis. However, only has trastuzumab, which is a monoclonal antibody targeting ErbB2, been recently approved as the first molecularly targetd drug against gastric cancer. The PI3K/Akt pathway is one of the most important signaling pathways in human carcinogenesis. In the present study, a high prevalence of *PIK3CA *amplification was found in gastric cancer, which was significantly associated with poor prognosis of gastric cancer patients. Importantly, *PIK3CA *amplification could aberrantly activate the PI3K/Akt signaling pathway. In addition, the drugs, such as mTOR and Akt inhibitors that target this signaling pathway, are being vigorously developed [36]. Thus, for some gastric cancer patients harbored oncogenic alterations within the PI3K/Akt signaling pathway, such as *PIK3CA *amplification, combination therapy with an mTOR or Akt inhibitor should be considered.

## Conclusions

In summary, our data showed that *PIK3CA *mutations may not be frequent genetic event in gastric cancer, however, *PIK3CA *gene was highly amplified in gastric cancer. To our knowledge, the present study is the first to demonstrate that *PIK3CA *amplification was significantly associated with poor survival in gastric cancer. More importantly, *PIK3C*A amplification was closely associated with elevated p-Akt, suggesting that this genetic alteration may be a major mechanism in activating the PI3K/Akt signaling pathway, and contribute to gastric tumorigenesis. Thus, specific genotype-based targeting against the PI3K/Akt signaling pathway may be an effective therapeutic strategy for gastric cancer.

## Competing interests

The authors declare that they have no competing interests.

## Authors' contributions

PH conceived and designed the experiments. JS, DY, WL, NW, HL, and LX performed the experiments. DY, YC, and NH collected the patient materials. MJ, NH, and PH analyzed the data. HL and GZ carried out the histopathological analysis. BS and PH contributed reagents/materials/analysis tools. JS and PH wrote the paper. All authors are in agreement with the content of the manuscript and this submission.

## Pre-publication history

The pre-publication history for this paper can be accessed here:

http://www.biomedcentral.com/1471-2407/12/50/prepub

## Supplementary Material

Additional file 1 **Figure S1**. Somatic mutations identified in the *PIK3CA *gene in gastric cancers. Examples of somatic mutations found in the helical and kinase domains of PIK3CA. Arrows indicate the position of the missense mutations. The amino acid changes are given above the arrows. **Figure S2**. Association of *PIK3CA *mutations and amplification with poor survival in gastric cancer. Kaplan-Meier survival curves was made according to the presence of *PIK3CA *mutations or amplification in a large cohort of gastric cancers. (**A**) *PIK3CA *mutations were not associated with poor survival of the patients. (**B**) The patients with *PIK3CA *amplification had a significantly shorter survival than the patients without *PIK3CA *amplification (*P *= 0.01). *PIK3CA *Mu, *PIK3CA *mutations; *PIK3CA *Am, *PIK3CA *amplification; +, harboring *PIK3CA *mutations or amplification; -, the lack of *PIK3CA *mutations or amplification. **Table S1**. *PIK3CA *mutations and amplification in gastric cancer--univariate associations with clinicopathological features (OR† and 95%CI). Click here for file
