# Supplementary material for: Highly frequent PIK3CA amplification is associated with poor prognosis in gastric cancer
Source: BMC Cancer. 2012 Feb 1;12:50. doi: 10.1186/1471-2407-12-50 (PMC3299648; doi:10.1186/1471-2407-12-50)
Supplement: Additional file 1 — Figure S1. Somatic mutations identified in the PIK3CA gene in gastric cancers. Examples of somatic mutations found in the helical and kinase domains of PIK3CA. Arrows indicate the position of the missense mutations. The amino acid changes are given above the arrows. Figure S2. Association of PIK3CA mutations and amplification with poor survival in gastric cancer. Kaplan-Meier survival curves was made according to the presence of PIK3CA mutations or amplification in a large cohort of gastric cancers. (A) PIK3CA mutations were not associated with poor survival of the patients. (B) The patients with PIK3CA amplification had a significantly shorter survival than the patients without PIK3CA amplification (P = 0.01). PIK3CA Mu, PIK3CA mutations; PIK3CA Am, PIK3CA amplification; +, harboring PIK3CA mutations or amplification; -, the lack of PIK3CA mutations or amplification. Table S1. PIK3CA mutations and amplification in gastric cancer--univariate associations with clinicopathological features (OR† and 95%CI). [file 1471-2407-12-50-S1.DOC]

**Additional file**

**Figure legends**

**Figure S1. Somatic mutations identified in the *PIK3CA* gene in gastric cancers.** Examples of somatic mutations found in the helical and kinase domains of PIK3CA. Arrows indicate the position of the missense mutations. The amino acid changes are given above the arrows.

**Figure S2. Association of *PIK3CA* mutations and amplification with poor survival in gastric cancer.**  Kaplan-Meier survival curves was made according to the presence of *PIK3CA* mutations or amplification in a large cohort of gastric cancers. (**A**) *PIK3CA* mutations were not associated with poor survival of the patients. (**B**) The patients with *PIK3CA* amplification had a significantly shorter survival than the patients without *PIK3CA* amplification (*P* =0.01). *PIK3CA* Mu, *PIK3CA* mutations; *PIK3CA* Am, *PIK3CA* amplification; +, harboring *PIK3CA* mutations or amplification; -, the lack of *PIK3CA* mutations or amplification.

**Table S1**. *PIK3CA* mutations and amplification in gastric cancer―univariate associations with clinicopathological features (OR† and 95%CI)

| **Factors** | *PIK3CA* mutations [OR† (95% CI)] | *PIK3CA* amplification [OR† (95% CI)] |
| --- | --- | --- |
| Male *vs.* Female | 2.14 (0.48―9.63) | 1.11 (0.46―2.70) |
| Age^1^ | 0.99 (0.50―1.96) | 0.89 (0.63―1.26) |
| Tumor size^2^ | 0.83 (0.34―2.02) | 0.97 (0.61―1.52) |
| Differentiation^3^ | 5.05 (0.60―42.5) | 1.92 (0.92―4.01) |
| Tumor invasion^4^ | 0.86 (0.35―2.13) | 1.15 (0.68―1.92) |
| Tumor stage^5^ | 0.96 (0.45―2.07) | 0.97 (0.65―1.47) |
| Lymph node metastasis | 1.03 (0.23―4.53) | 1.46 (0.69―3.06) |
| No. of LNM^6^ | 0.76 (0.32―1.81) | 1.35 (0.87―2.09) |
| Survival status^7^ | 0.59 (0.13―2.59) | 2.57 (1.21―5.48)* |

† OR: odds ratio with 95% confidence interval

1 Age (per 10 years)

2 Tumor size (≤3 cm; >3cm and ≤5 cm; >5)

3 Differentiation (well or moderate; poor or no differentiation)

4 Invasion depth (T1; T2; T3; T4)

5 Tumor stage (I; II; III; IV)

6 No. of LNM (lymph node metastasis) (0; 1-6; 7-15; >16)

7 Survival status (alive; dead)

* Significant at *P* <0.05


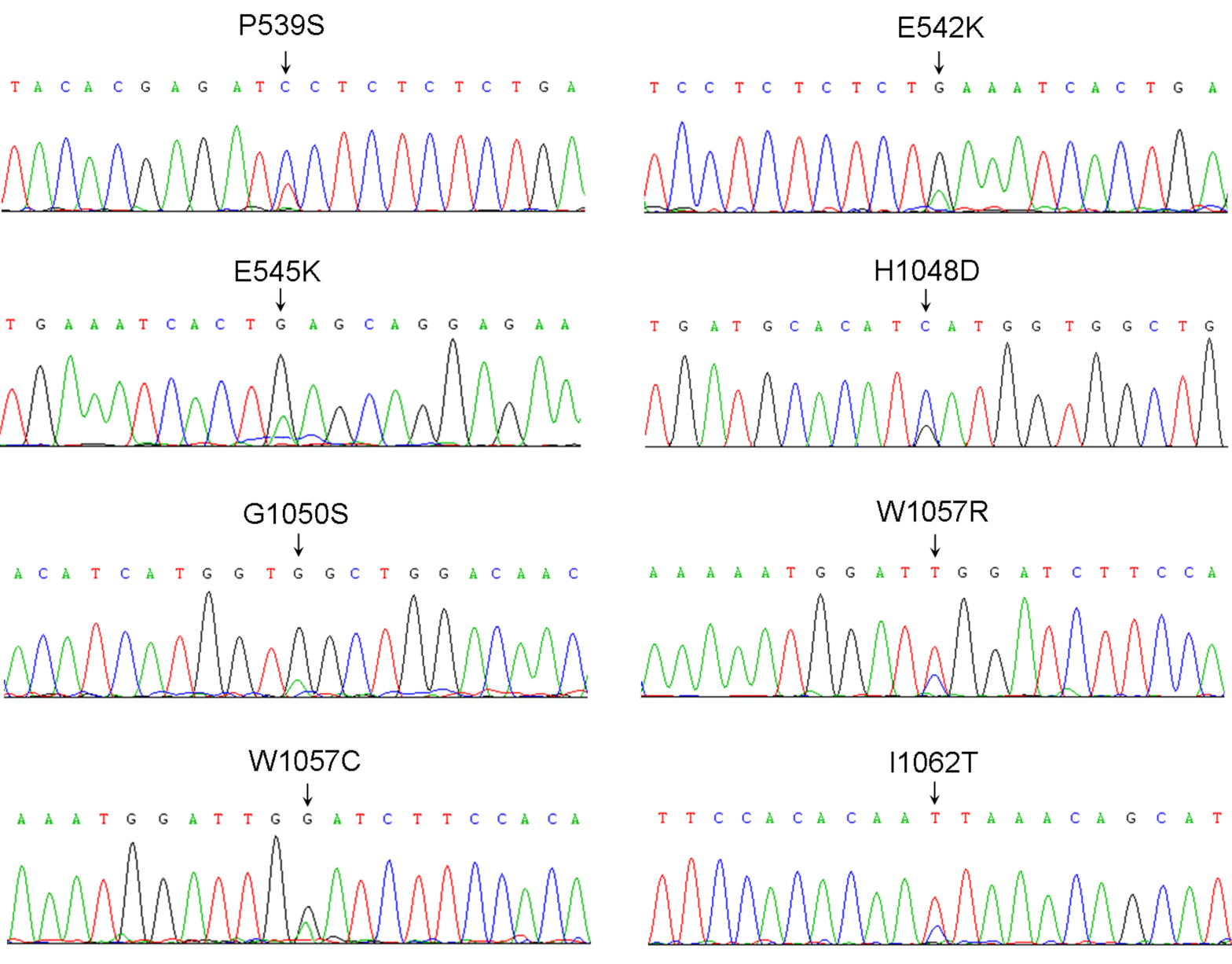


**Jing Shi, et al., Figure S1**

**
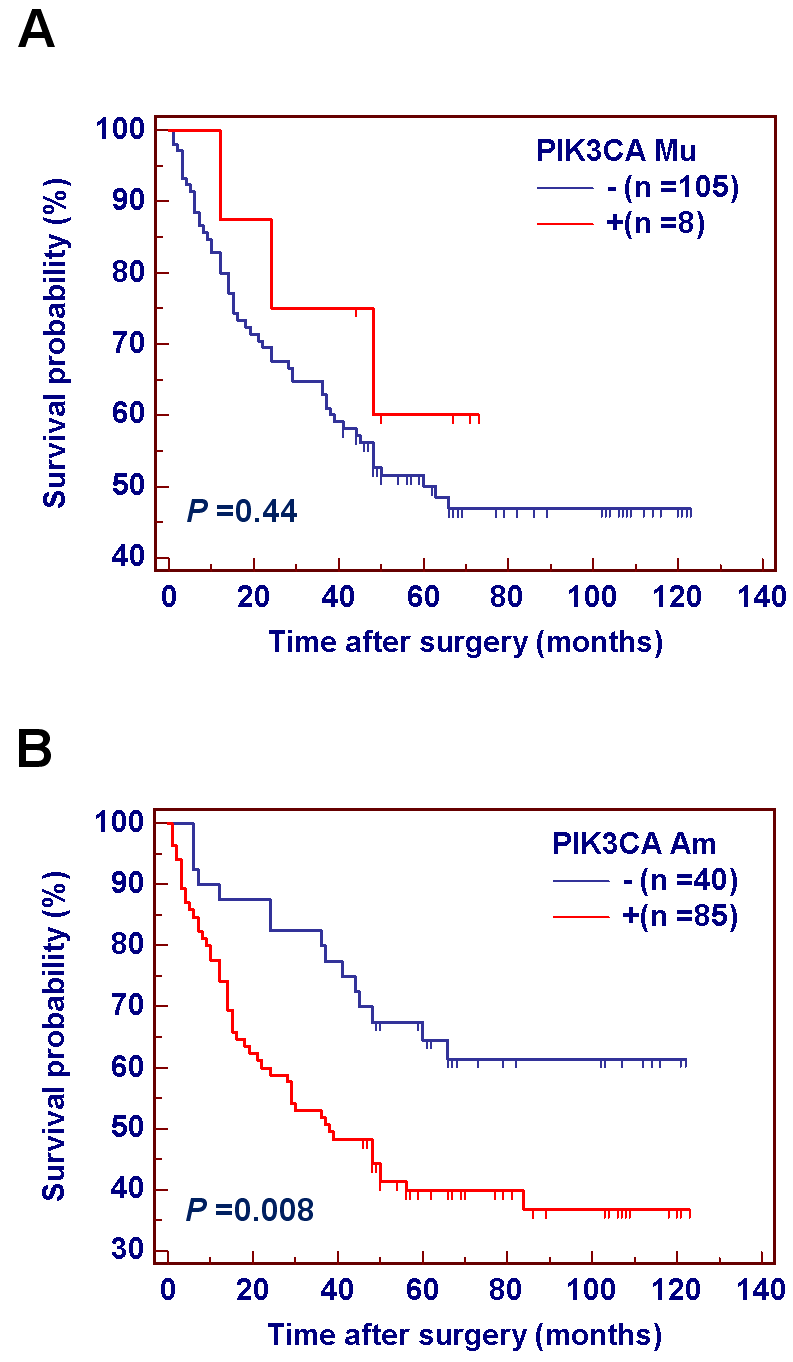
**

**Jing Shi, et al., Figure S2**
